# Innovative Metal-Organic Frameworks for Targeted Oral Cancer Therapy: A Review

**DOI:** 10.3390/ma16134685

**Published:** 2023-06-29

**Authors:** Seyyed Mojtaba Mousavi, Seyyed Alireza Hashemi, Fatemeh Fallahi Nezhad, Mojtaba Binazadeh, Milad Dehdashtijahromi, Navid Omidifar, Yasamin Ghahramani, Chin Wei Lai, Wei-Hung Chiang, Ahmad Gholami

**Affiliations:** 1 Department of Chemical Engineering, National Taiwan University of Science and Technology, Taipei 10607, Taiwan; mousavi.nano@gmail.com; 2 Nanomaterials and Polymer Nanocomposites Laboratory, School of Engineering, University of British Columbia, Kelowna, BC V1V 1V7, Canada; sa_hashemi@sums.ac.ir; 3 Biotechnology Research Center, Shiraz University of Medical Sciences, Shiraz 71439-14693, Iran; f.falahi1372@gmail.com; 4 Department of Chemical Engineering, School of Chemical and Petroleum Engineering, Shiraz 71557-13876, Iran; binazadeh@shirazu.ac.ir (M.B.); mdj.v.n3@gmail.co (M.D.); 5 Department of Pathology, Shiraz University of Medical Sciences, Shiraz 71439-14693, Iran; omidifarn@sums.ac.ir; 6 Associate Professor of Endodontics Department of Endodontics, School of Dentistry Oral and Dental Disease Research Center Shiraz University of Medical Sciences, Shiraz 71956-15787, Iran; ghahramani.yas@gmail.com; 7 Nanotechnology & Catalysis Research Centre (NANOCAT), University of Malaya (UM), Kuala Lumpur 50603, Malaysia

**Keywords:** metal-organic frameworks (MOFs), oral squamous cell carcinoma (OSCC), BioMOFs

## Abstract

Metal-organic frameworks (MOFs) have proven to be very effective carriers for drug delivery in various biological applications. In recent years, the development of hybrid nanostructures has made significant progress, including developing an innovative MOF-loaded nanocomposite with a highly porous structure and low toxicity that can be used to fabricate core-shell nanocomposites by combining complementary materials. This review study discusses using MOF materials in cancer treatment, imaging, and antibacterial effects, focusing on oral cancer cells. For patients with oral cancer, we offer a regular program for accurately designing and producing various anticancer and antibacterial agents to achieve maximum effectiveness and the lowest side effects. Also, we want to ensure that the anticancer agent works optimally and has as few side effects as possible before it is tested in vitro and in vivo. It is also essential that new anticancer drugs for cancer treatment are tested for efficacy and safety before they go into further research.

## 1. Introduction

Cancer threatens human health and causes millions of deaths annually [1,2]. In 2018, 354 thousand oral cavity and lip cancer cases were registered worldwide, and 177 thousand people died from the disease [3]. In medicine, oral cancer is defined as developing tumor cells in the mouth, lips, tongue, hard and soft palate, buccal mucosa, mandibular and maxillary alveolar ridges, posterior deltoids of molars, and oral cavity [4].

Oral squamous cell carcinomas (OSCC) make up over 90% of all cases of oral cancer, commonly occurring in middle-aged and older populations. Public statistics indicate there will be 377,000 new oral cavity cancer cases and 177,000 new deaths from this disease worldwide in 2020. Over the past three decades, mortality and morbidity rates for OSCC have not changed significantly, although treatment methods have improved dramatically in recent years. The probability that a patient with OSCC will survive five years or longer is usually 40% to 50% [5,6,7,8,9,10]. Head and Neck Squamous Cell Carcinoma (HNSCC) is treated with radiation therapy, surgery, or both simultaneously [11,12]. Outcomes in patients with advanced disease are still suboptimal, even with a combination of surgery and radiotherapy. Therefore, chemotherapy is now used to improve the treatment outcomes of patients with HNSCC and to preserve the organs of these patients [13]. Chemotherapy remains the most popular form of treatment. However, due to inherent limitations, such as unfavorable side effects, poor pharmacokinetics, and poor biodistribution, it is no longer practical to directly administer therapeutic drugs to patients in the usual way [1]. The leading cause of OSCC treatment failure and the main side effect of chemotherapy is the accumulation of chemotherapeutic drugs in normal tissues because they accumulate easily [14]. In addition, the use of combination therapies, which increase the risk of life-threatening side effects, is crucial when there is resistance to single therapies. In addition, several anticancer drugs cannot be administered systemically, including radioisotopes, toxins, nucleic acids, and hydrophobic drugs. Chemotherapeutic chemicals can be engineered to work more efficiently against cancer cells while not accumulating in healthy tissues for optimal efficacy—the development of targeted drug delivery using nanocarriers to circumvent the above limitations. Selective targeting improves therapeutic efficacy, concentration in the tumor site, drug solubility, and bioavailability [15,16].

In recent decades, various nanomaterials, including organic polymers and inorganic porous materials, have been successfully developed for cancer therapies and bioapplications [3,17]. However, either lower loading capacity or uncontrolled release limits their applicability. Recently, a new approach has been investigated to overcome these problems, namely the use of MOFs as carriers for biological purposes [3].

Metal-organic frameworks (MOFs), a hybrid inorganic-organic material, comprise metal ions or clusters coordinated with organic ligands to create structures. The organic ligands are referred to as “linkers,” and the inorganic metal clusters are referred to as “secondary-building units” [3,18]. The structure and properties of MOFs are determined mainly by their organic bonding partners and metal ions. MOFs can be synthesized with most metal ions. MOFs are used in various industries, including catalysis, sensing, drug delivery, imaging of biological samples, molecular sieves, gas storage, separation, and imaging. In addition, porous MOFs are generally physiologically stable, possess variable pore structure and large surface area, good thermal stability, high drug loading, tunable pore diameter, sustained release, functionality, and excellent biocompatibility, making them suitable for the functionalization of specific biomolecules in situ or post-synthesis, either directly on metal surfaces or using organic ligands [19].

The potential of MOFs to prevent or treat bacterial infections that may contribute to the development or progression of oral cancer could be investigated, as bacteria have been linked to the development of oral cancers and can worsen the effects of chemotherapy and radiation therapy. If MOFs exhibit antibacterial activity, they could be used as an adjunct therapy to reduce bacterial load and improve treatment outcomes. MOFs could also serve as drug delivery vehicles to target oral cancer cells specifically while providing antibacterial activity to prevent infection [20]. Therefore, the antibacterial properties of MOFs could be a crucial factor in their potential application in oral cancer cell research and therapy [21,22]. According to a study, certain bacteria in the oral cavity can contribute to developing oral cancers. The most Roswell-confirmed bacteria are *Fusobacterium nucleatum* and *Porphyromonas gingivalis* [23]. Other bacteria linked to oral cancer include *P. melanogenic*, *Capnocytophaga gingivalis*, *Capnocytophaga ochracea*, *Eubacterium*, *Aggregatibacter segnis*, *Prevotella intermedia*, *Catonella morbi* and *Peptostreptococcus stomatitis* [24,25]. *Streptococcus thermophiles* and *Streptococcus mitis* have also been found to produce acetaldehyde, a known carcinogen [26].

In this review, the application of MOFs in the treatment of oral cancer is discussed. In addition, the applications and antibacterial properties of MOFs are described.

## 2. Metal-Organic Frameworks

**Figure 1:**
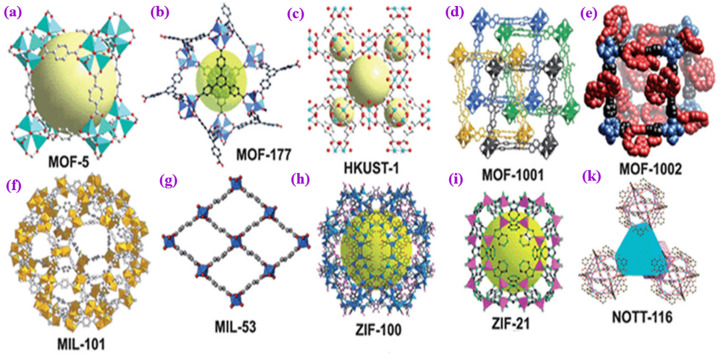
Illustration of some MOF structures. (**a**) MOF-5 is a three-dimensional framework structure composed of terephthalic acid and metal cluster Zn4O [57]. (**b**) MOF-177 is a three-dimensional framework structure composed of octahedral Zn_4_O(−COO)_6_ and triangular 1,3,5-benzenetribenzoate (BTB) units [58]. (**c**) The HKUST-1 material is a type of MOF comprising copper nodes and benzene-1,3,5-tricarboxylate linker molecules [59]. (**d**) MOF-1001 has a non-interpenetrating cubic structure isoreticular to MOF-5 [60]. (**e**) MOF-1002 shares an identical cubic backbone with MOF-1001 [61]. (**f**) MIL-101(Cr) is a MOF that is formed by the coordination of a Cr3O ionic cluster with terephthalic acid (H2BDC) [62,63]. (**g**) MIL-53 is a three-dimensional framework structure composed of inorganic [M-OH] chains connected to four neighboring inorganic chains by terephthalate-based linker molecules [64]. (**h**) ZIF-100 is a zeolitic imidazolate framework (ZIF). The structure of ZIF-100 is crystalline and porous, with tetrahedral networks that resemble those of zeolites [65,66]. (**i**) ZIF-21 is a zeolitic imidazolate framework (ZIF) arisopologically isomorphic with zeolites. The structure of ZIF-21 is composed of tetrahedrally coordinated transition metal ions (Zn, Fe, Cu, C, e.g.) connected by imidazolate linkers [67]. (**k**) NOTT-116 is a metal-organic polyhedral framework that consists of two types of cages: a truncated octahedron (cage A) with six square windows and eight hexagonal faces and a truncated tetrahedron (cage B) with four triangular windows and four hexagonal faces [68,69]. Copyright © 2023, MDPI.

MOFs are crystalline porous solids constructed by stitching together inorganic polynuclear clusters known as secondary building units (SBUs) and organic linkers via strong bonds. Based on the results of studies conducted in recent years, metal-containing SBUs are primarily finite units in which the junctions where they are connected to linkers form geometric shapes with well-defined geometric shapes, such as octahedra and squares, which are well-defined geometric shapes [27,28]. (See Figure 1). If designed by expanded zeolite topology, MOFs’ inorganic and organic structures would have larger pores and higher porosity than zeolites [29].

The link length between inorganic SBU is due to high porosity and longer organic ligands benefitting large pore diameter [29,30,31,32]. There are more than 20,000 MOF structures worldwide [33,34]. In most cases, MOFs are synthesized by either solvothermal or hydrothermal synthesis methods. The reaction is initiated by small-scale electrical heating, which takes several hours to days. Alternative synthesis methods not only shorten the synthesis time but are also capable of producing smaller and more uniform crystals. These include microwave-assisted [35], sonochemical [36], electrochemical [37], and mechanical synthesis methods [38], which can speed up synthesis and result in smaller, more homogeneous crystals. An essential feature of functional metal-organic frameworks is that they can be endowed with various functions that can be used for multiple purposes. Overall, the most effective synthesis method must be investigated to meet the unique requirements of biomedical applications, considering the effects of surface functionalization on the chemical properties, morphology, and size of MOFs. Precise regulation of porosity and particle size, as well as the effects of surface attraction on their metabolic activities in vivo, present MOFs with critical challenges in biological applications. Various surface functionalization techniques offer practical ways to increase physiological and colloidal stability, create specialized entities for drug-controlled release and precisely targeted identification, enhance catalytic reactivity, and extend circulation time [4].

A significant advantage is that these functions can be incorporated into MOF structures, metal ions or clusters, and organic ligands as bridging elements. For example, the organic ligand groups (such as -N_3_, -NH_2_, and -COOH) and the metal nodes on the surface of MOFs can be conjugated by covalent bonds or strong coordination with the pendants used for surface functionalization [39]. To increase the colloidal/physiological stability and decrease the immune response of MOFs, pendant polymers such as liposomes and polyethylene glycol (PEG) are commonly used [40,41].

Biomacromolecules, such as peptides and nucleic acids, bind to the surface of MOFs via coordination bonds, which gives them the capability for target identification, analytical detection, drug delivery, and bioimaging [42]. Significantly, supramolecular interactions immobilize supramolecular macrocycles on the surface of MOFs to reduce side effects and control drug release during drug delivery [4,43]. The stability of MOFs in most biomedical applications is one of the critical issues to be considered in their use.

Due to the enormous diversity of MOF structures, it is difficult to make generic statements about the stability of MOFs. Molecular dynamics models and tests have shown that the IRMOF-1 framework (isoreticular metal-organic framework) collapses at water contents of 3.9% and above due to the oxygen atoms of water replacing the oxygen atoms of IRMOF-1 in the Zn coordination shells [44,45].

**Figure 2:**
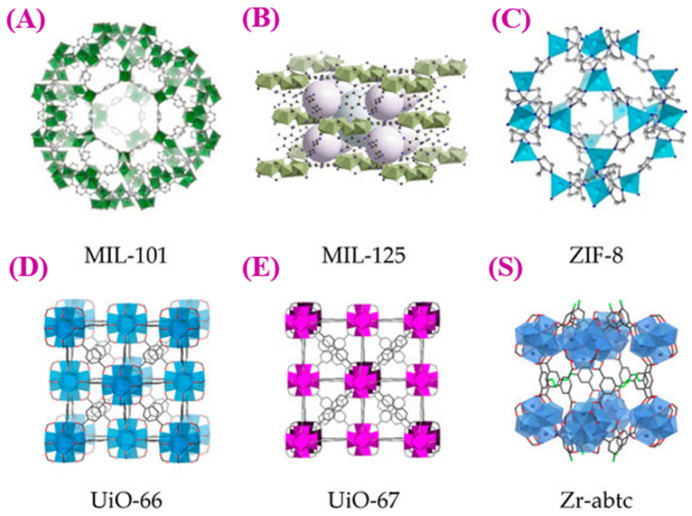
The figure shows different types of MOFs with excellent thermal and chemical stability. (**A**) MIL-101(Cr) is comprised of trimeric chromium(III) octahedral clusters [63]. (**B**) MIL-125 consists of Ti_8_O8-(OH)4-(O_2_C-C_6_H_5_-CO_2_)_6_ elementary units with a quasi-cubic tetragonal structure [70]. (**C**) ZIF-8’s basic structural unit comprises a Zn atom and four imidazolate linkers with a tetrahedral structure [71]. (**D**) UIO-66 comprises zirconium oxide clusters bridged by terephthalic acid ligands [72]. (**E**) UIO-67 is created by joining 12 inorganic subunits with inorganic octahedral Zr6 units [73]. (**S**) Zr-abtc consists of 8-connected Zr_6_(μ3-O)_4_(μ3-OH)_4_(COO)_8_ SBUs that are propagated by 4-connected abtc4− linkers along three dimensions [74,75]. Copyright © 2023, MDPI.

Recently, significant progress has been made in developing water-stable MOFs [46]. Several examples of MOF structures are relatively stable, despite the general perception that MOFs have limited stability. In particular, zeolitic imidazolate frameworks (ZIFs), zeolite-like MOFs (ZMOFs), and metal-azolate frameworks (MAFs, ZIF-57), combining azolate and carboxylate coordination capabilities demonstrate substantially improved hydrothermal and chemical stability [47,48,49]. ZIF-8 exhibits high hydrothermal stability as it lacks polar groups in its pore apertures, making it hydrophobic. MIL-type MOF consists of a lanthanide component (Y, Tb, Eu) or a trivalent metal component (Fe, Al, Cr), which forms strong bonds with oxygen-anion-terminated linkers and exhibits high chemical stability [50,51]. The hydrothermal stability of various MOFs was investigated at different temperatures and pressures during steaming. Hydrothermally stable MOFs include zirconium-based UiO-66, MIL-14, and hydrophobic ZIFs [52]. MOF mechanical stability depends on the structural features and valence of inorganic components. Due to the high strength of their inorganic-linker connection, MOFs based on divalent metals, for instance, have more excellent mechanical stability and are consequently more resilient to crystal breakage and deformation [53,54] (See Figure 2). One of the studies aimed to synthesize pores decorated with pyrazine on a zinc MOF to give it water stability and bio-friendliness. It was shown that a new porous MOF containing Zn(II) ions could be prepared by combining 2,3,5,6-tetrakis (4-carboxyphenyl) pyrazine (H4tcpp) in combination with metal Zn(II) ions. A study was conducted to show that pyrazine functionalized linkers (2,3,5,6), when used in combination with 4,4′-bipyridine (by) as an auxiliary ligand, can interfere with metal-Zn(II) ions by disrupting their electrostatic interactions. According to one study, Ni-CPO-27 dissolved very little after spending days in bovine serum. Fewer than 10% of MOF was dissolved in serum after four days. According to studies by Horcajada et al., Fe-MIL-101 and Fe-MIL-100 (MIL: Materials of the Institut Lavoisier) [55] remain stable in biological fluids without losing their effectiveness as drug carriers over extended periods [44,56].

A recent study has shown that nanocarriers are remarkably effective in simultaneously achieving high drug concentrations, controlled drug delivery, and low cytotoxicity. The results confirm that this is indeed the case.

## 3. Biological Metal-Organic Frameworks (BioMOFs)

A new class of MOFs has been discovered, called BioMOFs, combining MOFs’ chemistry with the biology of living organisms [76,77]. The terms used to describe BioMOFs are not clearly defined. Several different viewpoints have been expressed in the literature. According to some experts, BioMOFs are classes of very porous MOFs that have a variety of uses in biology and medicine. In contrast, others contend that BioMOFs are a class of highly porous MOFs used in many fields across biology and medicine. In the former case, MOFs are characterized by biomimetic units derived from nature in their composition [76,78,79,80,81]. Biological applications are the focus of this latter area of research. It is, therefore, worthwhile to note that there are two primary categories of BioMOFs. (1) BioMOFs that make use of Active Pharmaceutical Ingredients (APIs) as building blocks for constructing the framework, thereby avoiding the necessity of large pores, and (2) BioMOFs that make use of APIs as guests for constructing the framework from localized resources [82,83]. As an example of an API or any other bioactive organic molecule, this subsection of the BioMOF class utilizes endogenous molecules to construct the framework [84,85]. Bio-MOFs have diverse structures and functions due to the variety of possible interactions between biological ligands and metal centers [86,87]. Consequently, endogenous organic linkers have been found to exhibit this property, especially those based on amino acids. In particular, this approach seems particularly useful in using ligands with therapeutic activity derived from inorganic polytopic compounds, such as Zn-MOFs prepared from phenylalanine and tyrosine derivatives [88,89]. Several parameters must be considered when designing BioMOFs, including the application, the risk versus benefit balance, the biodistribution, the degradation kinetics, the accumulation in tissues, organs, and the excretion [90,91]. As a result of MOF synthesis, drugs can be delivered using either exogenous (do not interfere with the body’s cycles) or endogenous (constituting a part of the body’s composition) linkers, although experiments with the former seem to be more prevalent [44,92]. Additionally, it is crucial to note that by directly using a therapeutic molecule as a linker, there is no need for large pores to be created. The release of the drug can be achieved through the degradation of the solid in a way that does not result in any side effects resulting from the release of a non-active ligand [89,90]. The use of bioMOFs in biological applications has recently been proposed in the last few years, mainly for delivering active ingredients (AIs) in controlled environments [80,93]. As a controlled delivery system, BioMOFs are particularly suitable due to several specific properties. The advantages of this technology include (1) porosity no longer being a problem as the scaffolds are degraded, resulting in the release of active ingredients and bioactive molecules; (2) multi-step synthesis is not required as the molecules are incorporated into the matrix itself; (3) it is possible to investigate the synergistic effects of the metal and the active chemical; (4) co-delivery of drugs may be possible when a porous network is formed with one ingredient A, and another ingredient is added. An effective targeted drug delivery method, which can be achieved by using porous bioMOF structures, can be performed by simultaneously delivering different drugs, including those bound to the pores of the structure and those that are part of the framework, as part of a less toxic, effective, and more targeted drug delivery method [80,82].

## 4. Metal-Organic Frameworks (MOFs) for Biomedical Applications

Since their unique properties attract growing interest in many biomedical fields, MOFs and their composites have received much attention. These properties include their biodegradability, high loading capacity, and adaptability, as well as their application as cargo delivery agents for cancer treatment (nucleic acids, drugs, dyes, and proteins), bioimaging, antimicrobial agents, biosensing, and biocatalysis [94,95]. Consequently, MOF precursors must be biocompatible to contribute to the bioavailability of the overall system [96]. MOFs used in biomedical applications generally comprise metals essential to the body, such as iron, zinc, or magnesium [96,97,98,99]. Recent technological advancements have led to significant progress in the biomedical applications of MOFs. These advancements include the (1) encapsulation, protection, and delivery of goods, as well as (2) biomedical imaging and the use of (3) therapeutic agents [100]. MOFs are among the widely used materials offering various therapeutic agent strategies. Following advances in MOF synthesis methods, it is now possible to fabricate MOFs with specific functionality by carefully designing topology, metal clusters, and organic linkers. With the encapsulation of therapeutic chemicals, combination therapies can be realized thanks to the porosity of MOFs, which allows interaction between external species and active sites in the MOF structure. Four common strategies for incorporating therapeutics into MOF carrier systems are using therapeutics as linkers, non-covalent encapsulation, conjugation with the linkers, and attachment to the SBUs [101,102].

To achieve drug delivery, nanocarriers must be designed to alter drug hydrophilicity, ensure controlled delivery, and prevent drugs from binding nonspecifically to irrelevant molecules. In addition, medications can be absorbed and excreted differently to achieve this [100,103,104]. Bioimaging using MOFs involves using these porous materials to encapsulate imaging agents for improved contrast and sensitivity in medical imaging. MOFs have potential as antibacterial agents due to their ability to release metal ions with antimicrobial properties. They can also be used to release drugs in a controlled manner, which can improve efficacy and reduce the side effects of the drugs [105,106,107,108,109]. In this regard, this section will discuss the biological applications of MOFs.

### 4.1. Bioimaging

Detecting and evaluating the therapeutic effect of a drug carrier is one of the most challenging aspects of drug administration [110]. In recent years, a growing number of studies have demonstrated the efficacy of molecular imaging in the noninvasive assessment of pharmacokinetics and healing when contrast agents are combined with molecular imaging [110]. MOFs have emerged as an emerging imaging agent in recent years due to their variety of structures, sizes, and metallic compound nodes, making them an attractive option for enhancing signals at targeted sites and increasing their contrast. The Enhanced Permeability and Retention (EPR) effect increases the likelihood that these nanoparticles will accumulate in tumor sites when combined with molecules that target tumors. In tumorous tissue, the absence of vascular supporting tissue indicates the formation of pores and leaky vessels (with a diameter of 100 nm to 2 μm), and the poor lymphatic system provides a fantastic chance for the treatment of cancer; this phenomenon is known as the EPR effect [111]. In the context of imaging a variety of diseases, including cancer, using a wide range of imaging modalities, including computed tomography (CT), fluorescence imaging (FL), positron emission tomography (PET), and magnetic resonance imaging (MRI), MOFs have proven to be extremely sensitive and flexible imaging materials [100]. These materials’ extremely high sensitivity and flexibility have proven extremely useful for imaging. Another advantage of MOFs, including those modified for intracellular imaging, is that they can be fabricated with various fluorophores that can be used for intracellular imaging.

For visually guided photodynamic/photothermal treatment (PDT/PTT), Zhu et al., 2018 published a study on a nano theranostics platform made of a nanoscale metal-organic iron porphyrin scaffold. The bovine serum albumin (BSA), sulfonamides (SAs), and BSA/SAs modification of nMOF resulted in NPs with longer circulation time in blood and increased accumulation at the tumor site. Due to the organic porphyrin ligands and magnetic iron ion centers, the prepared BSA/SAs-nMOF NPs served as MRI agents and actively targeting to tumor cells [112,113]. 

Steunou and coworkers recently described a mesoporous superparamagnetic magnetic composite material composed of nanoscopic maghemite particles for fabricating high-resolution magnetic resonance imaging based on superparamagnetic mesoporous magnetic MIL-100 (Fe) composite materials. Since the composite was fabricated under physiological conditions, it showed good MRI stability and capability [55]. Moreover, the relative relaxometry value of MIL-100 (Fe) was nine times higher when the NPs were on the order of 10 wt% Fe_2_O_3_ of the total NPs content. This reversal effect was caused by the very high saturation magnetization of the composite material. As well as the composite material was found to have excellent antitumor activity and biocompatibility after being loaded with Doxorubicin (Dox). Due to its high biocompatibility and antitumor activity, it was also found to be a potential candidate for an MRI contrast agent [4,55,114].

**Figure 3:**
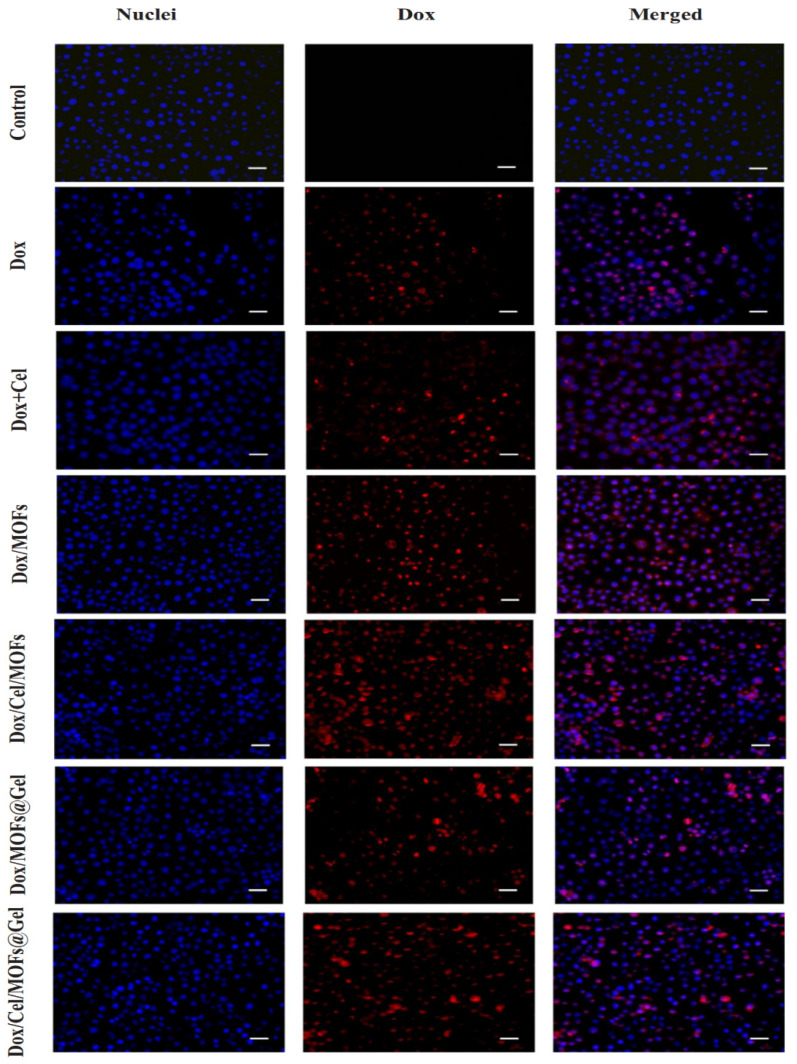
The fluorescence micrographs show SCC9 cells incubated for 4 h with complete medium, free Dox, free Dox + Cel, free Dox/MOFs, free Dox/Cel/MOFs, free Dox/MOFs@Gel, and free Dox/Cel/MOFs@Gel (scale bar: 100 m) [115]. Copyright © 2023, Elsevier.

In another study, Tan et al. developed Dox/Cel/MOFs@Gel nano vehicle for oral cancer therapy. To determine whether the obtained system can adapt to oral cancer cells (squamous carcinoma cells (SCC-9)) and to determine cellular uptake, fluorescence microscopy experiments were performed on these cells. The study aimed to determine if the system could enter the cells as predicted. A variety of Dox materials were used in this study. These included free Dox materials, Dox + Cel materials, Dox/MOFs, Dox/Cel/MOFs, and Dox/Cel/MOFs@Gel materials, in addition to the free Dox materials. After four hours of culturing the cells in SCC-9 cells, the red fluorescence of Dox was visible around the nucleus. It was obvious that blue fluorescence could be seen around the nucleus after four hours of culturing the cells, indicating that the cells had internalized Dox after four hours of culturing. Comparison of the uptake of free Dox in the Dox + Cel group with the uptake of Dox in the free Dox group revealed no significant differences in the uptake pattern between the two groups, indicating that the addition of Cel did not affect the uptake of free Dox in any way. Free Dox/MOFs and free Dox/Cel/OFs all had stronger intracellular fluorescence than free Dox, implying that MOFs may promote the internalization of Dox-loaded systems more effectively than those containing free Dox (Figure 3) [115].

**Figure 4:**
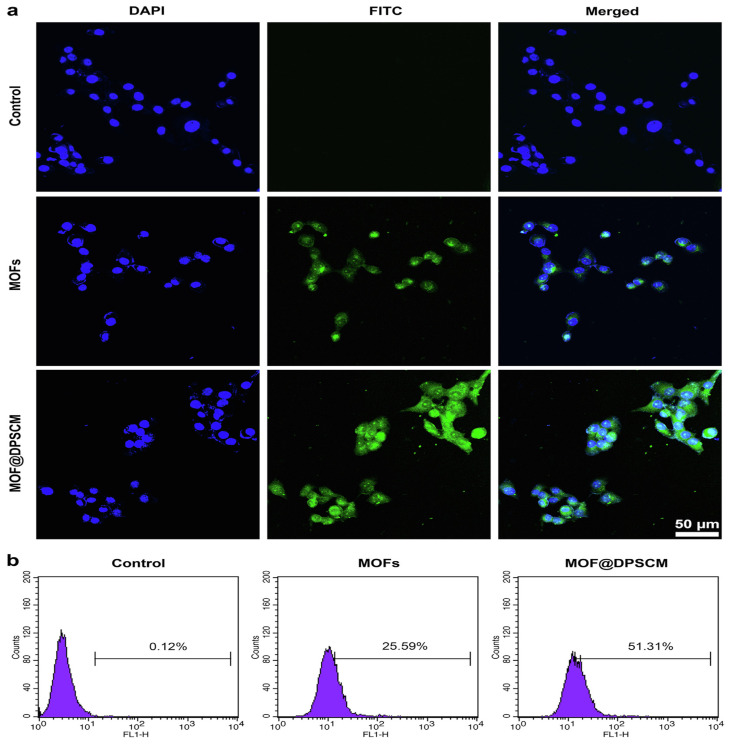

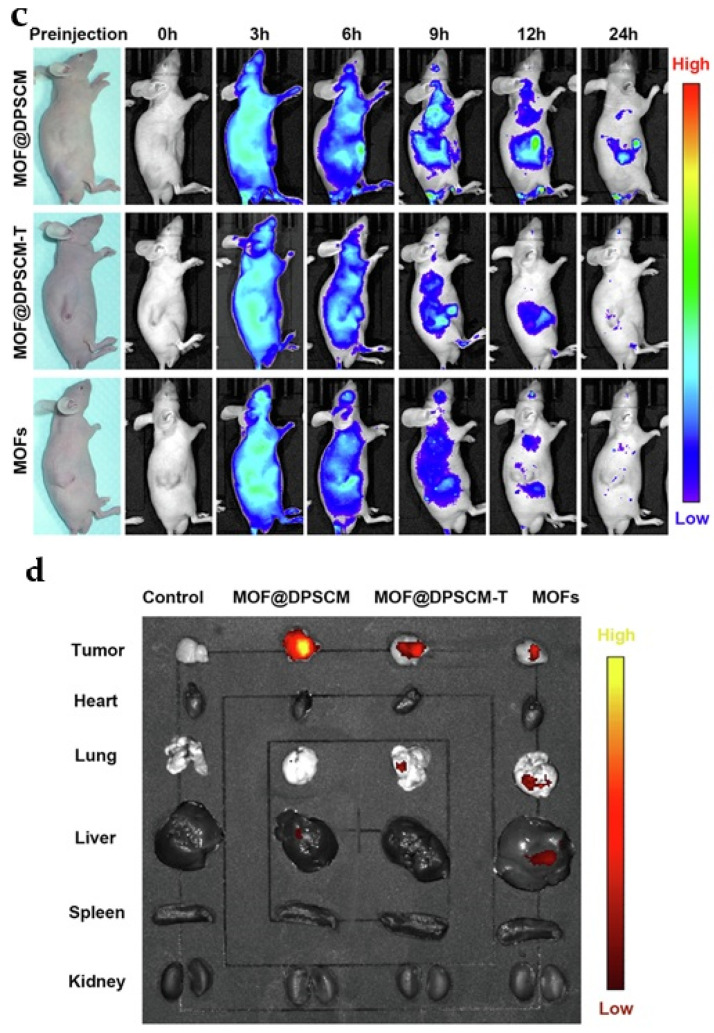
CAL27 cells are used to compare internalization between MOFs and MOF@DPSCM. (**a**) Confocal scanning light microscopy (CSLM) images. (**b**) Analysis using flow cytometry of CAL27 cells treated with MOF@DPSCM or FITC-labeled MOFs. As a control, cells were treated without any nanoparticles. Biodistribution of MOFs, MOF@DPSCM-T, and MOF@DPSCM in vivo. (**c**) After various intervals of time, intravenous administration of MOFs, MOF@DPSCM-T, and MOF@DPSCM labeled with Cy7, animals with CAL27 tumors were photographed in vivo in real-time with live fluorescence. (**d**) Ex vivo images of the animals in the central tissues of (**a**). As a control, the mice received PBS injections [116], Copyright © 2023, Elsevier.

Zhou et al. performed in vitro and in vivo tests in their investigation after the MOF-DOX@DPSCM nanocarrier was developed to cure OSCC cancer. Fluorescence microscopy assays on the cells were used to determine how well the manufactured nanocarrier was internalized. The dental pulp mesenchymal stem cells (DPSCs) showed an intriguing innate ability to settle in the tumor, and their cell membranes could produce MOF@DPSCM to target OSCC both in vitro and in vivo. Considering what the internalization experiments showed, only a limited amount of FITC-labeled MOF@DPSCM adhered to or penetrated CAL27 cells. The flow cytometry and fluorescence results show that the surface of MOFs with DPSC membrane shells improves the internalization of the cells into CAL27 cells and promotes selective binding specifically. The results of the in vivo assays also proved that the MOF@DPSCM had a unique OSCC targeting capacity, as they were still present in the OSCC and CAL27 tumors 24 h compared to the control groups following injection. Figure 4 shows that after the intravenous injection of MOFs, MOF@DPSCM-T, and MOF@DPSCM tagged with Cy7, real-time fluorescence pictures of mice with CAL27 tumors were taken at various periods [116].

### 4.2. Antibacterial

In today’s era of antibiotic abuse, the spread of bacterial infections is a public health concern and contributes to increased mortality and morbidity rates worldwide. Therefore, we must seek even more effective and novel methods to eradicate bacteria [55]. Recently, studies have been conducted on MOFs combining organic and inorganic scaffolds to create an antibacterial compound [94]. MOFs are believed to possess antibacterial properties because they contain metal ions that can be readily taken up by bacteria and disrupt the synthesis of proteins by incorporating the metal ions into the bacterial cell wall, thus disrupting the functions of the bacterial cell. Several recent publications have pointed out the efficacy of MOFs against *Escherichia coli*, including MOF-199 and CuBTC (copper(II)-benzene-1,3,5-tricarboxylate) as antibacterial agents [117,118]. Based on the structure of the cell walls of bacteria, or the way they divide their cell wall into two parts, they can be classified as Gram-negative and Gram-positive bacteria. Gram-negative and Gram-positive bacteria are distinguished by a thicker peptidoglycan layer and a species-specific outer membrane at the end of the peptidoglycan layer [119,120]. The outer membrane of Gram-negative bacteria covers the peptidoglycan layer. In contrast, Gram-positive bacteria have a thin peptidoglycan coating on their cell walls [121]. Penicillin, rifampicin, chloramphenicol, tetracycline, and lincomycin, some of the antibiotics commonly used to treat bacterial infections, are known for their antibacterial activity. These bacteria cannot only interfere with synthesizing cell walls, DNA, RNA, and proteins [122]. Although these mechanisms have evolved, it is essential to remember that bacteria have developed various ways to resist them. These include altering antibiotic targets, modifying or destroying antibiotics, reducing antibiotic accumulation by decreasing permeability or enhancing efflux pumps, and other strategies. As a result of adverse side effects such as nephrotoxicity and ototoxicity, the drug does not appear to be as effective as expected. Due to these side effects, the drug’s efficacy was significantly impaired, contrary to expectations [119].

**Figure 5:**
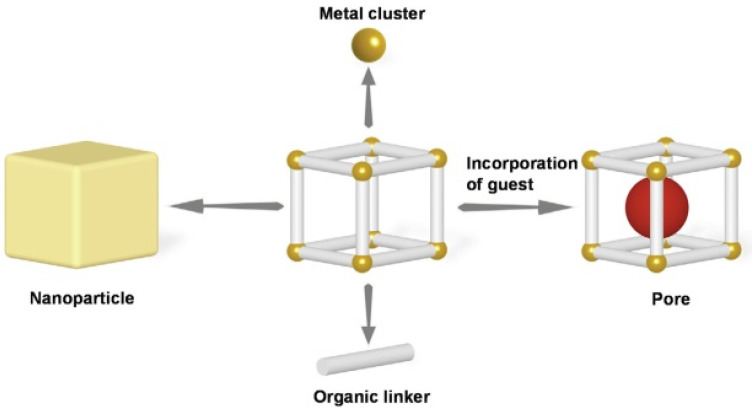
Antimicrobial MOF applications utilize four structural elements to achieve effective antimicrobial activity [123]. Copyright © 2023, Elsevier.

Designing desired antibacterial properties for practical applications involves understanding the structures and properties of MOFs, which has primarily focused on (i) examinations of a particular metal’s activity; (ii) examples of the biological activity and adaptability of organic ligands, including reversible adsorption and desorption of guest molecules as well as ligand functional groups that provide sites for post-synthesis and host-guest interactions.; (iii) sizes, the particular geometric configurations, surface chemistry, surface charges, and catalytic properties of MOF NPs; (iv) pores with an extremely high specific surface area and porosity that it loads a variety of guests [123]. (See Figure 5).

**Figure 6:**
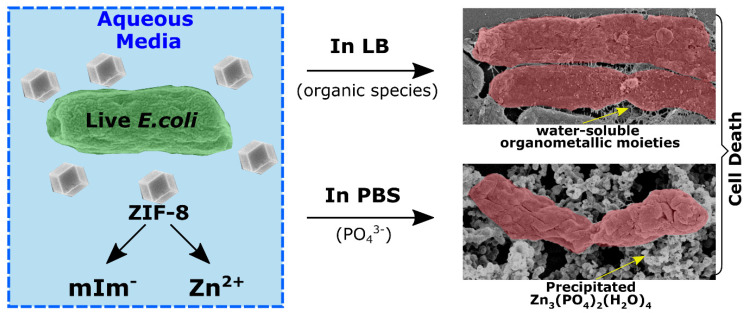
Schematic illustration of how ZIF−8 combines with PBS to create the antimicrobial Zn_3_(PO_4_)_2_(H_2_O)_4_ [129], Copyright © 2023, Elsevier.

MOFs, similar to metal nanoparticles, are thought to be able to store and release ions, acting as an ion reservoir that releases ions in a controlled manner [124,125]. The most likely cause of MOFs’ antibacterial and biotoxic properties is the release of the metal from the bulk of the framework, either in the form of a cation or as tiny pieces of the MOF framework. By assuming that the antibacterial activity is directly proportional to the ease of cation release, which depends on the MOF structure’s relative hardness, Berchel et al. [126] provided an intriguing strategy. They believed the antibacterial action directly related to how simple it was to release cations, which depended on the MOF structure’s rigid structures. Based on Pearson’s “hard and soft (Lewis) acids and bases” (HSAB) hypothesis, the authors argued that the ease of cation release depends on the relative hardness of the cations (Lewis acids) and organic linkers (Lewis bases) that make up the MOF. When a rigid base and a soft acid are linked, the entire structure has moderate stability, making it more susceptible to deterioration and more likely to release cations. The pairings of complex acids and hard bases and soft acids and soft bases should be used to construct the most stable structures [127]. When MOFs react with bacterial cell wall components, these ions are continuously released into the environment and act as focal sources of toxic ions. There is evidence that MOFs may exhibit antibacterial properties due to the degradation of organic ligands and metal ions within them and the synergistic effect of these two phenomena [76,79]. It was found that several properties of MOFs can exert antibacterial effects due to the sustained release of their organic ligand. Besides the metal ions that MOFs release, there is a possibility that the antibacterial properties of the organic ligand may also play a role in this synergistic effect. MOFs have been suggested to possess antibacterial properties similar to metal ions because they release metal ions [127,128]. These include releasing reactive oxygen species (ROS), changes in ionic equilibrium, interactions with thiol groups of proteins, inactivation of critical enzymes, damage to cells, photothermal effect, physical contact, oxidative stress, metal ions, and ligands [128]. Taheri et al. studied the degradation of ZIF-8 in organisms to learn more about how released ions destroy bacteria in the physiological environment. When immersed in phosphate-buffered saline (PBS), ZIF-8 first released Zn^2+^ and then immediately formed Zn_3_(PO_4_)_2_ (a broad-spectrum antibacterial agent), simulating the physiological environment. (Figure 6). The formation of Zn_3_(PO_4_)_2_ in the physiological environment was suggested to be the reason for the potent antibacterial activity of ZIF-8 [129].

Aguado et al. prepared two Co-based MOFs (ZIF-67 and Co-SIM-1) using 2-methylimidazole and 4-methyl-5-imidazole carboxaldehyde as organic ligands, respectively. At concentrations of ZIF-67 and Co-SIM-1 between 5 and 10 mg/L, it was shown that *E. coli*, *P. putida*, and *S. cerevisiae* all exhibited growth inhibition more significantly than 50%. They also linked these MOFs’ sustained and low release of Co^2+^ to their significant antibacterial activity [130]. According to a recent study, MOFs with metal components of Cu, Mg, Mn, Fe, Co, Zn, or Ni and 2,5-dihydroxy terephthalate as organic linkers showed antibacterial activity against bacteria, which is an impressive result. Research with MOFs containing Ni and Zn, among other metals, has demonstrated that one has promising antimicrobial properties [131,132]. MOFs possess antibacterial properties due to the presence of metal ions that can interact with the cell walls of bacteria and alter the process of protein synthesis to ensure that antibacterial properties are maintained. Many metal oxide films with promising antibacterial properties have been in the past, including HKUST-1, MOF-199, and CuBTC. In the literature, Wang et al. reported that a new CuMOF [CuC_12_H_14_N_2_O_8_] based on 4,4-dicarboxy-2,2-bipyridic acid (H_2_dcbp) was prepared under different reaction conditions with identical structures exhibiting four different morphologies (blocky layer, disk-like layer, lumpy layer, and sage-like layer) [133].

Recently, certain MOFs with two-dimensional morphologies were prepared, which showed excellent antibacterial properties due to the physical interaction between the materials and bacteria. Yuan et al. synthesized ZIF-L with a nano-dagger surface, which is entirely different from the dodecahedral structure of ZIF-8. According to the results, ZIF-L displayed excellent antibacterial activity (logarithmic decrease >7 for *S. aureus* and *E. coli*) and long-lasting bactericidal activity (further than four times application). They suggested that the surface of ZIF nano-daggers can effectively destroy germs by physical contact [134]. Table 1 briefly overviews the antibacterial applications of metal-organic framework composites and the details of the experiments and formulations.

### 4.3. Drug Delivery System

In recent decades, inorganic carriers such as carbon structures, nanoparticles of mesoporous silica, nitrides, and oxides, and organic vehicles such as micelle, dendrimers, and polymers have been developed for the delivery of anticancer agents and drugs to oral cancer cells to improve the bioavailability and therapeutic effect of the drug [144]. Still, inorganic systems’ metabolic mechanisms, biological distribution, and immunogenicity urgently need further systematic investigation. On the other hand, using organic materials as drug carriers are often associated with poor loading and premature drug leakage [19]. Compared to the materials mentioned above, MOFs have emerged as one of the most encouraging options to solve most of the difficulties associated with drug shipment. Various drugs can be encapsulated in MOFs, including those with hydrophilic, hydrophobic, and amphiphilic properties [4].

**Figure 7:**
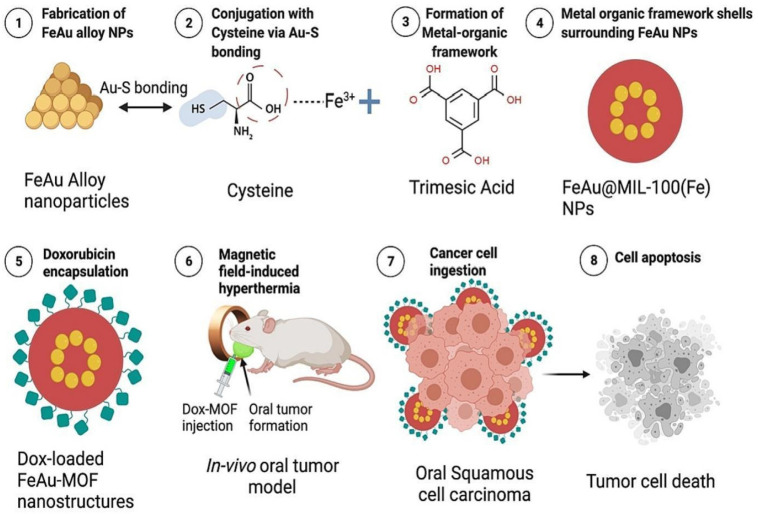
For the therapy and imaging of cancer caused by hyperthermia, metal-organic framework-FeAu NPs nanostructures were designed. Cysteine was used as a linker to create various MOF shells. An increase in the number of MOF shells improved doxorubicin encapsulation and biocompatibility. In an in-vivo mouse model, Dox-loaded MOF-FeAu nanostructures showed a significant decrease in tumor growth, an improvement in survival, and helped with tumor imaging [145]. Copyright © 2023, Elsevier.

In a study by Dhawan et al., the hydrophobic drug Dox was encapsulated in a nanocarrier (FeAu@MIL-100). To fabricate FeAu@MOF nanostructures, nanoparticles of the FeAu alloy (FeAu NPs) were first prepared and then coated with MIL-100 (Fe) MOFs. They encapsulated Dox into the nanostructures and assessed how the platform worked for imaging and cancer theranostics. FeAu@MOF nanostructures exhibited superparamagnetism when stimulated with magnetic hyperthermia behavior and an alternating magnetic field (AMF) and showed Dox release and encapsulation efficiencies of 97.19 and 69.95%, respectively. AMF-induced hyperthermia resulted in the death of 90% of HSC-3 cells of OSCC according to in vitro assays, suggesting its potential use in cancer theranostics. Finally, FeAu@MOF nanostructures in an in vivo mouse model enhanced imaging contrast, decreased tumor volume by 30 times, and tumor weight by 10 times, enhancing cumulative survival and demonstrating the potential of this method for the treatment of oral cavity cancer [145] (See Figure 7).

**Figure 8:**
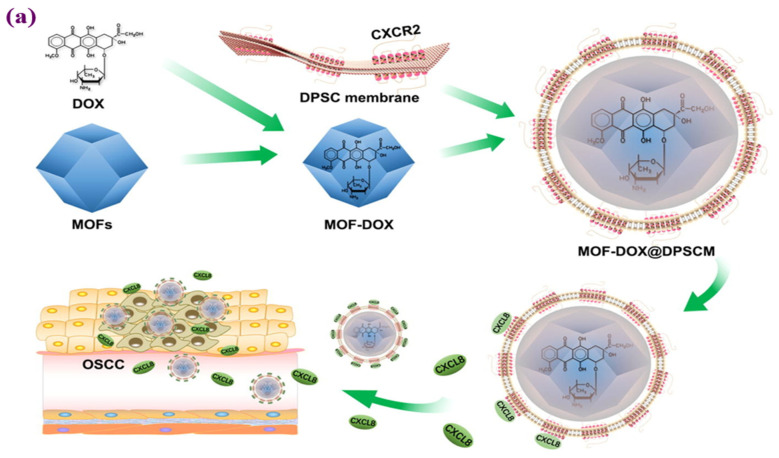

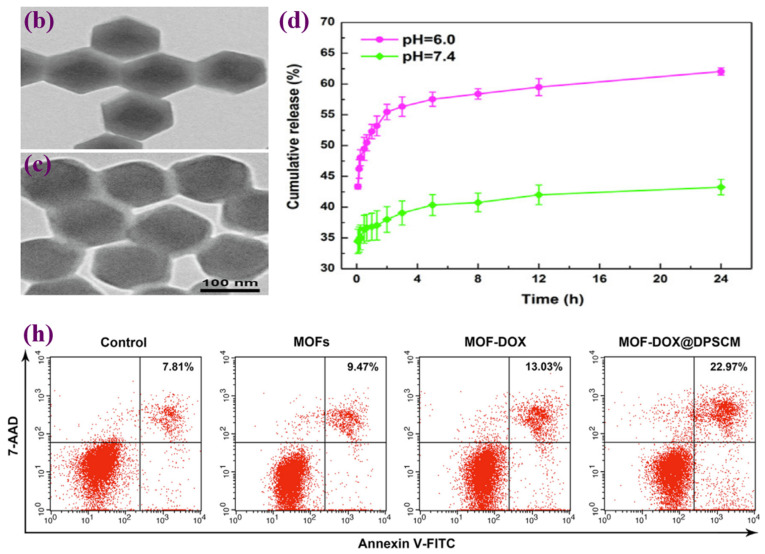
Figure illustrating the (**a**) modification of MOFs by (DPSC) membranes for the treatment of OSCC. Characteristics of MOFs and MOF@DPSCM. (**b**) TEM images of MOFs. (**c**) TEM images of MOF@DPSCM. (**d**) Release experiment of Dox from MOF-Dox@DPSCM under pH 6.0 and 7.4 necrosis and apoptosis assays. (**h**) MOFs, MOF-Dox, and MOF-Dox@DPSCM, effects on CAL27 cells in an in vitro assay [116], Copyright © 2023, Elsevier.

In another study, Zhou [116] and his colleagues developed MOF nanoparticles coated with membranes derived from dental pulp mesenchymal stem cells (DPSC) containing the CXCR2 receptor. It has been shown that MOF-Dox@DPSCM can carry Dox and be chemoattracted by chemokines such as CXCL8 released from OSCC, reducing OSCC rate growth in vitro and in vivo. Figure 8 shows a schematic of the application of DPSC membranes in conjunction with MOFs as a method for targeting OSCCs using MOFs. The release was measured at an acidic pH of 6.4. Flow cytometric analysis revealed that MOFs alone induced an apoptosis rate of 9.47% in CAL27 cells. In contrast, MOFDox@DPSCM induced 22.97% apoptotic CAL27 cells after 4 h of coculture. According to these experiments, the Dox in MOF-Dox@DPSCM effectively killed CAL27 and OSCC cells (Figure 8) [116].

In another study, Zn^2+^ and the hydrophilic drug disulfiram (DSF) were used to inhibit ALDH1+ cancer stem cells (CSCs). They developed an innovative metal-organic scaffold (IRMOF3)-Zn^2+^, and DSF was incorporated into the IRMOF3. Then folic acid (FA) was loaded onto the surface, resulting in IRMOF3 (IRMOF3-DSF-FA) for drug delivery. The IRMOF3-DSF-FA showed excellent metal ion loading capacity, high biocompatibility, and robust cell uptake capacity, which can target tumor tissues, deliver metal ions, and inhibit ALDH1+ CSCs. IRMOF3 could significantly interfere with the growth rate of tumors and CSCs-DSF-FA in in vivo studies, with no apparent adverse effects on major organs in treating oral cancer [146].

The encapsulation of molecules is enabled by non-covalent bonds between the molecules and MOFs, and this non-covalent bond allows molecules to be encapsulated by the pores of the MOF when their size is smaller than that of the pores and to take up molecules when their size is smaller than that of the pores. There is evidence that MOFs can be highly loaded with drugs using the non-covalent approach, as shown by the high loading of ibuprofen and cisplatin, the controllable release profile, and the absence of any burst response to the drug [100]. It has also been shown that using mesoporous silica (MPS) and zeolites in MOF structures results in strong scaffolds without a burst release effect. A MOF-based drug release mechanism differs from other nanocarriers in that the drug molecules are released slowly and with a controlled pattern. In contrast, different nanocarriers tend to release molecules in bursts [10,147,148]. Sun and colleagues developed an example using chiral nanoporous MOFs [(CH_3_)_2_NH_2_]_2_[Zn(TATAT)_2/3_]-3DMF-H_2_O. A hexadentate ligand (5,5′5″) of 1,3,5-triazine-2,4,6-triyl)triisophthalate (TATAT) and Zn^2+^ was developed to use Zn^2+^ as a hexadentate ligand and Zn^2+^ as a method for the delivery of 5-fluorouracil as an anticancer drug [149].

In an experimental study by An et al., four types of Bio-MOFs were evaluated for their drug loading and release ability (Bio-MOF-4, Bio-MOF-1, Bio-MOF-102, and Bio-MOF-100). In all Bio-MOF groups, the initial explosive release was followed by a progressive release over a prolonged time, ranging from 49 to 80 days [150].

The development of a polymer-coated MOF gatekeeper system responsive to enzymes was enabled in a study by using PCN-224 nano MOF and hyaluronic acid (HA) as substrates. NanoMOFs can form a stable surface coating containing carboxylic acids when exposed to HA. Multiple Zr clusters form on their outer surfaces, serving as gatekeepers for HA to create an array of coordination bonds between the contained carboxylic acids. In the following study, HA-Dox-PCN, Dox-PCN in the presence of HA-Dox-PCN, and Dox-PCN alone were applied to HeK293T, MDAMB-231, and SCC-7 cells without the presence of HA-Dox-PCN. It was found that the level of reactive oxygen species (ROS) in these cells could be measured using a BD FACSVerse flow cytometer. The researchers demonstrated that the HA-Dox-PCN nanoparticles could be directly targeted to cancer cells expressing the CD44 receptor, showing a selective effect of these nanoparticles on these cancer cells. This indicates that the nanoparticles can catalyze the uptake of their impact by the cancer cells, suggesting that they can induce their effects through catalysis. When administered to cells, the cellular uptake of HA-Dox-PCN and clathrin-mediated endocytosis correlate. Confocal microscopy and flow cytometry (FACs) were used to confirm that ROS was formed in the cells. Due to light irradiation, HA-Dox-PCN produced ROS in the SCC-7 and MDA-MB231 cells [151].

**Figure 9:**
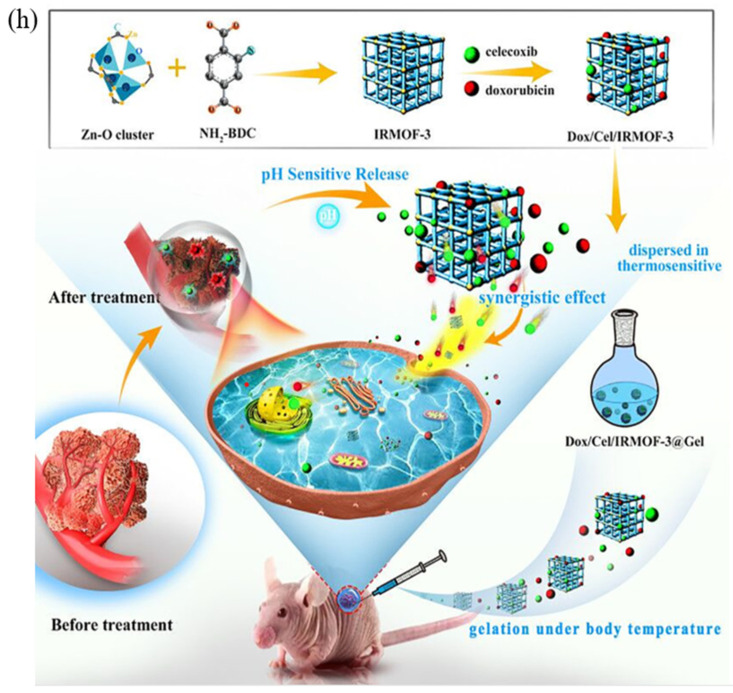

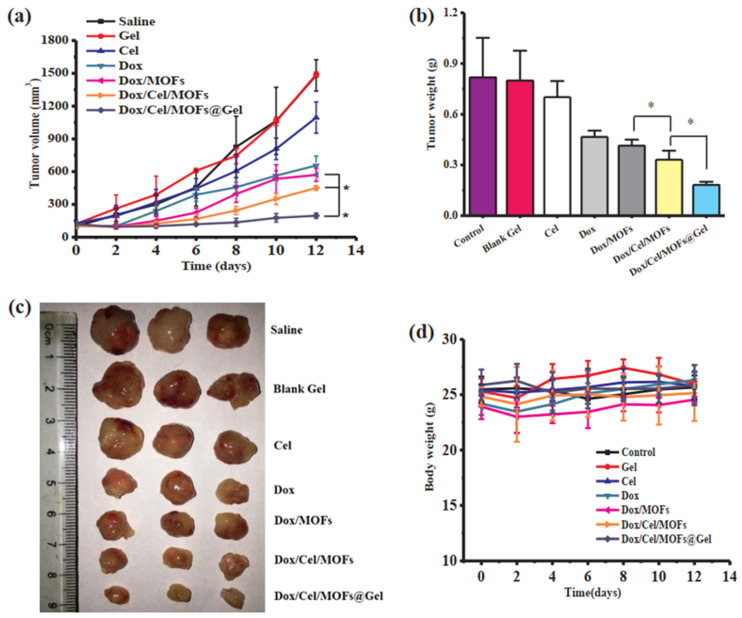
Schematic illustration of (**h**) a novel injectable metal-organic scaffold@thermosensitive hydrogel for local dual drug delivery during oral administration, Dox/Cel/MOFs@Gel Cancer Therapy. (**a**) Tumor volume change across all groups over 12 days. (**b**) The weight of the mice’s tumors after 12 days in each group. (**c**) An image of the tumors that were removed on day 12. (Data are expressed as the mean ± SD, * *p* < 0.05). (**d**) A measurement of mice’s average body weight taken 12 days after dosing [115], Copyright © 2023, Elsevier.

Local cancer therapy using several drugs is a revolutionary treatment strategy for enhancing therapeutic molecules’ persistent administration that successfully reduces tumor growth. One of the potential advantages of MOFs is the co-loading properties of medicines. In one study, Tan et al. developed a hybrid nanocomposite using thermosensitive hydrogels and metal-organic frameworks (MOFs) to create an injectable implant. To treat locally advanced oral cancer, doxorubicin and celecoxib were coloaded into the system (Dox/Cel/MOFs@Gel). In the lab, utilizing several kinds of oral cancer cells, including SCC-9 and KB, it was discovered that the platform could absorb a sizable amount of the medication and release it uniformly and pH-dependently. The researchers used a cell line containing free Dox and various Dox formulations (free, free + Cel, Dox/MOFs, Dox/Cel/MOFs, and Dox/MOFs) to study oral cavity cancer cells. It was found that Dox/MOFs and Dox/Cel/MOFs had different intracellular fluorescence than free Dox and Dox/MOFs, indicating that MOFs may improve the internalization of the Dox-loaded system in comparison to free Dox (Figure 9) [115]. The synergistic actions of Cel and Dox led to the nanocomposites’ exceptional tumor suppression effectiveness in vivo, which resulted in tumor death and controlled tumor angiogenesis [146].

**Figure 10:**
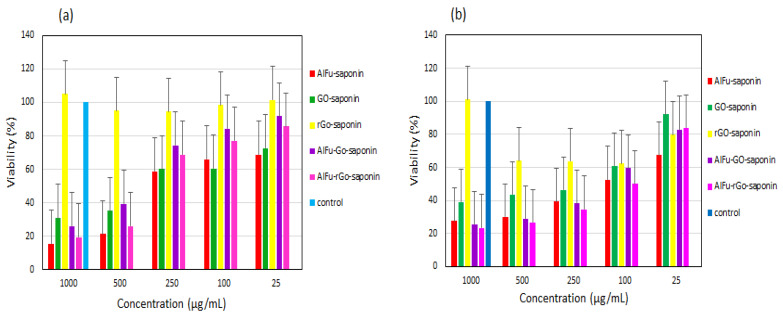
Use of AlFu (MOF)/GO/rGO for drug toxicity analysis and effects on the viability of PDL cells and OSCC with the combination of saponin and AlFu (MOF)/GO/rGO. Nanoparticles (MOFs) with saponin (**a**) on PDL cells. Nanoparticles (MOFs) with saponin (**b**) on OSCC cells [125]. Copyright © 2023, MDPI.

A study on GO/rGO doped with carboxylic acid doped with AlFu as a natural anticancer agent containing well-ordered saponin. GO carboxylic acid-containing/rGO was delivered to periodontal ligament fibroblasts (PDL) and OSCC with excellent PDL and OSCC GO results. To develop an innovative hybrid nanostructure with high porous properties and nontoxic to saponin loading, it was necessary to use aluminum fumarate (AlFu) as the core-shell nanocomposite of MOF. This study investigated nanocomposites containing aluminum fumarate as an integral component. AlFu modifies graphene oxides (GOs) and reduced graphene oxides (rGOs) in the core-shell nanocomposites. These compounds were determined to be anticancer agents against these cancer cells using two in vitro assays, including flow cytometry and MTT analyses. According to the MTT results, cells from PDL, exposed to AlFu-GO-saponin 250 mg/mL, survived between 74.46 and 16.02%, while cells from OSCC cells exposed to similar concentrations viability between 38.35 and 19.9%, proving that the nanostructure exhibited anticancer activity (Figure 10). The AlFu-rGO-saponin group displayed more apoptotic cells than the AlFu-GO-saponin group. The untreated group showed higher apoptotic cells than the AlFu-rGO-saponin group [125,152].

After applying various nanostructures to PDL and OSCC cell lines, flow cytometry was performed to determine whether the nanostructures had any adverse effects after being applied to the cells. In addition, Mousavi and colleagues observed whether the nanostructures induced apoptosis in the cells after the nanostructures were used. The results of the experiments show no statistically significant variation between the control group and the treated group in terms of the degree of cell apoptosis in the treated group, which is consistent with the results of the control group. According to the study results, OSCC cells show a significant difference compared to PDL cells regarding the effects of the control group (2.52/0.78%). It was found that both AlFu-GO-saponin groups and AlFu-rGO-saponin groups show an increase in apoptotic cells compared to the control groups. Both groups show an increase in apoptotic cells of 10.98 × 2.36–26.90 × 3.24% and 15.9 × 4.08–29.88 × 0.41%, respectively. In addition to the differences between the Saponin, AlFu-GO, and AlFu-rGO groups in terms of apoptotic cells, there were also some significant differences in the percentages of apoptotic cells between the Saponin, AlFu-GO, and AlFu-rGO groups, with a rate of 13.94% being the highest, 22.75% is the lowest, and 16% being the lowest in the Saponin, AlFu-GO, and AlFu-rGO groups, respectively. In addition to a closer look at these graphs, it is clear that the AlFu-rGO-saponin combination therapy was associated with a significant increase in apoptosis compared with the nanostructures alone; thus, nanoparticles alone did not achieve the same results as the combination (Table 2) [125].

Multifunctional nanomedical MOF platforms offer broad perspectives for imaging-assisted combination therapy in precision medicine for cancer. As an innovative nanomedicine cancer platform for combined cancer therapy with MRI-guided magnetically triggered chemotherapy and hyperthermia, Zhu et al. developed novel porous Fe_3_O_4_@C nanocomposites based on metal-organic frameworks (MOFs). By using this platform in mice with CAL27 tumors, it was found that the combination therapy of chemotherapy and magnetic hyperthermia significantly slowed down the growth of the tumor. The underlying process showed that the high cell death rate of the tumor, together with necrosis and a low microvessel density, inhibited tumor growth. There is much potential for the clinical treatment of cancer with this innovative nano-platform because it is easy to fabricate, releases drugs on demand, and enables magnetically triggered, highly efficient combination therapy of chemotherapy and magnetic hyperthermia [153].

Another MOF that has been used as a targeted nanocarrier with hyaluronic acid (HA) is the PCN-224 MOF. DOX-PCN NPs with a HA coating have been developed for dual chemo-photodynamic treatment. The preparation of spherical PCN-224 NPs with a diameter of 100 nm was shown to result in tremendous cellular uptake. The carboxylic acid functional groups of Zr metal nodes and HA established coordination bonds, which resulted in the formation of a HA layer on the surface of the nanoparticles. The hyaluronidase of the cancer cell cleaved the polymeric shell that served as a gatekeeper, releasing Dox. It also possesses hydrophilic properties that contribute to colloidal stability. In this regard, compared with Hek 293T (human embryonic kidney cell 293, CD44-negative cells), for CD44-positive cells, such as MDA-MB-231 (human mammary gland or breast cancer cells) and SCC-7 (mouse squamous cell carcinoma), the developed system HA-DOX-PCN demonstrated toxicity and selective cellular uptake under light irradiation [151]. Other MOFs employed in the drug delivery system are listed in Table 3.

## 5. Conclusions and Perspective

MOFs have developed to the point where they can now be used in various biomedical technologies thanks to recent developments in material science and advanced materials. MOFs have attracted the attention of researchers working in the fields of cancer diagnostics, drug delivery, imaging, and biosensing, to mention a few, due to their adaptability in structure and function. With continued development and study, MOFs will soon be a viable option for usage as a single platform for numerous applications in biology and cancer therapy. One of the main problems of conventional cancer therapies is off-target effects on healthy tissue. Recently, thanks to the development of novel nanomaterials, targeted therapies for tumors have been developed that allow for more targeted cancer treatment with less harmful effects of chemotherapeutic agents on human cells. However, using nanomedicines in clinical practice has several drawbacks, including their reduced ability to overcome biological barriers and unfavorable off-target effects. Since MOF nanomaterials have advantages in imaging, drug delivery, and antibacterial properties, we focus on their use in cancer treatment in this review. There have been many experimental studies on these nanomaterials. Still, there are few clinical trials, so further clinical research is needed to prove the benefits and efficacy of these materials in the clinic. In addition, effective drug release and high drug concentration in target cells are required for clinical treatment; therefore, combination therapies must be used to improve the efficacy of MOFs. To achieve optimal results, it is also crucial that MOFs are carefully synthesized to allow most normal cells to survive during the in vitro and in vivo treatment process. Studies have shown that MOF nanomaterials, such as ZIF, AlFu, and MIL-100, perform exceptionally well in drug delivery. These materials have been proven in vivo and in vitro studies to have low toxicity and high surface area.

## Figures and Tables

**Table 1:** Synopsis for MOF composites with different antibacterial chemicals used in antibacterial applications [135]. Copyright © 2023, Elsevier.

S. No.	MOFs Composites	MOF’s Diameter	Antibacterial Compound	Kind of Bacteria	Combination Type	Antibacterial Activity	Reference
1	Zn50Co50-ZIF	100 nm	Zn50Co50-ZIF	*S. aureus*	one-pot method	Concentration = 1 mg/mL	[136]
2	Ceftazidime@ZIF-8	400 nm	Ceftazidime	*E. coli*	Mixture procedure		[137]
3	MIL-100 (Fe)@gentamicin	145–200 nm	gentamicin	*S. epidermidis* and *S. aureus*	simple impregnation procedure	*S. epidermidis*: MIC = 0.125 mg/L *S. aureus*: MIC = 0.5–1 mg/L	[138]
4	UiO-66/Poly(ε-caprolactone) MMMs		Poly(ε-caprolactone)/Mixed-matrix membranes	*E. coli*	Drawdown coating method	Standard plate count: Effectively inhibited within 30 min irradiation	[139]
5	Tool-box with a mixed MOF	<50 nm	Commercially plover membrane	*S. aureus* and *E. coli*	Sandwich multilayered membrane by casting	Standard plate count: Exhibited 5-log fold reduction within 15 min of incubation	[140]
6	CP/CNF/ZIF-67	10 nm	Carboxylated cellulose nanofiber	*E. coli*	In Situ on the fiber	Standard plate count: Antibacterial rate reached 80% ZOI = 12 mm	[141]
7	Ag-MOF functionalized TFC membrane		Polyamide membrane	*E. coli*	In Situ functionalization	Fluorescence microscopy: Bacterial mortality of approximately 100% was attained	[142]
8	Cu-BTC@silk fibers		Silk fibers	*S.aureus* and *E. coli*	layer-by-layer method	*S. aureus*: ZOI = 6.5 to 7.5 mm *E. coli*: ZOI = 7.7 to 8.0 mm	[143]

**Table 2:** The cell populations of OSCC and PDL cell lines undergoing early, late and cumulative apoptosis were examined in each group [125], Copyright © 2023, MDPI.

Compound	Late Apoptosis (%)		Early Apoptosis (%)		Cumulative Apoptosis (%)	
	PDL Cell Line	PDL Cell Line	OSCC Cell Line	OSCC Cell Line	PDL Cell Line	OSCC Cell Line
AlFu-rGO	11.25 ± 3.12	5.35 ± 0.58	7.66 ± 0.96	9.51 ± 0.51	16.60 ± 2.54	17.17 ± 2.80
AlFu-GO	8.79 ± 0.74	6.9 ± 3.39	5.69 ± 1.85	11.5 ± 1.33	15.69 ± 3.93	17.19 ± 2.26
AlFu-GO-saponin	7.13 ± 1.57	3.85 ± 0.07	11.30 ± 0.07	15.60 ± 0.16	10.98 ± 2.36	26.90 ± 3.24
AlFu-rGO-saponin	11.2 ± 1.43	4.7 ± 1.90	16.29 ± 3.48	13.59 ± 2.11	15.9 ± 4.08	29.88 ± 0.41
saponin	3.45 ± 0.41	11.51 ± 0.14	9.95 ± 1.05	12.80 ± 2.57	14.96 ± 3.04	22.75 ± 1.28
control	1.32 ± 0.08	1.20 ± 0.45	0.35 ± 0.81	0195 ± 0.31	2.52 ± 0.78	1.31 ± 0.62

**Table 3:** List of MOFs and their targeted agents employed in drug delivery systems [154]. Copyright © 2023, MDPI.

S. No.	MOFs	Drugs	Mechanism	Biological Test System	Reference
1	ZIF-8	Doxorubicin	Encapsulation	Breast cancer cell lines	[155]
2	ZIF-8	Ceftazidime	NA	*Escherichia coli*	[137]
3	MIL-100 (Fe)	Indocyanine green	π–π interaction	xenograft tumors/MCF-7 cells	[156]
4	MIL-100 (Fe)	Doxorubicin	NA	HepG-2 cells	[157]
5	MIL-100 (Fe)	Metformin hydrochloride	pH-cleavable bonds	PBS Buffer	[158]
6	MIL-101 (Fe)	BODIPY	NA	HT-29 human colon adenocarcinoma cells	[159]
7	MIL-101 (Fe)	Doxorubicin	NA	H-22 tumor-bearing mice	[160]
8	MOF-74 (Fe)	Ibuprofen	Ion exchange	PC12 cells	[161]
9	HKUST-1	Ibuprofen and guaiacol anethole	NA	PBS buffer	[162]
10	MIL-100 (Fe)	Doxorubicin	Host-Guest Interactions	Tris Buffer	[163]
11	NU-1000	Insulin	NA	Nucleic acids	[164]
12	NU-1000	Insulin	NA	PBS Buffer	[165]
13	Zn-MOF	5-Fluorouracil	pH-controlled	PBS Buffer	[166]
14	UiO-66@ Fe_3_O_4_	Doxorubicin	π–π interaction	HeLa, 3T3	[167]
15	UiO-66	Cisplatin	Encapsulation	U-87 MG cancer cell and HSC-3	[168]
16	UiO-68	Cisplatin	Encapsulation	SKOV-3 cells	[169]

## Data Availability

All data generated or analyzed during this study are included in this published article.
